# CT-Only Radiotherapy: An Exploratory Study for Automatic Dose Prediction on Rectal Cancer Patients *Via* Deep Adversarial Network

**DOI:** 10.3389/fonc.2022.875661

**Published:** 2022-07-18

**Authors:** Jiaqi Cui, Zhengyang Jiao, Zhigong Wei, Xiaolin Hu, Yan Wang, Jianghong Xiao, Xingchen Peng

**Affiliations:** ^1^ School of Computer Science, Sichuan University, Chengdu, China; ^2^ Department of Biotherapy, Cancer Center, West China Hospital, Sichuan University, Chengdu, China; ^3^ West China School of Nursing, West China Hospital, Sichuan University, Chengdu, China; ^4^ Department of Radiation Oncology, Cancer Center, West China Hospital, Sichuan University, Chengdu, China

**Keywords:** dose prediction, deep learning, radiotherapy planning CT-scan, rectal cancer, GAN structure

## Abstract

**Purpose:**

Current deep learning methods for dose prediction require manual delineations of planning target volume (PTV) and organs at risk (OARs) besides the original CT images. Perceiving the time cost of manual contour delineation, we expect to explore the feasibility of accelerating the radiotherapy planning by leveraging only the CT images to produce high-quality dose distribution maps while generating the contour information automatically.

**Materials and Methods:**

We developed a generative adversarial network (GAN) with multi-task learning (MTL) strategy to produce accurate dose distribution maps without manually delineated contours. To balance the relative importance of each task (i.e., the primary dose prediction task and the auxiliary tumor segmentation task), a multi-task loss function was employed. Our model was trained, validated and evaluated on a cohort of 130 rectal cancer patients.

**Results:**

Experimental results manifest the feasibility and improvements of our contour-free method. Compared to other mainstream methods (i.e., U-net, DeepLabV3+, DoseNet, and GAN), the proposed method produces the leading performance with statistically significant improvements by achieving the highest HI of 1.023 (3.27E-5) and the lowest prediction error with ΔD95 of 0.125 (0.035) and ΔDmean of 0.023 (4.19E-4), respectively. The DVH differences between the predicted dose and the ideal dose are subtle and the errors in the difference maps are minimal. In addition, we conducted the ablation study to validate the effectiveness of each module. Furthermore, the results of attention maps also prove that our CT-only prediction model is capable of paying attention to both the target tumor (i.e., high dose distribution area) and the surrounding healthy tissues (i.e., low dose distribution areas).

**Conclusion:**

The proposed CT-only dose prediction framework is capable of producing acceptable dose maps and reducing the time and labor for manual delineation, thus having great clinical potential in providing accurate and accelerated radiotherapy. Code is available at https://github.com/joegit-code/DoseWithCT

## Introduction

Rectal cancer is the third most deadly and fourth most commonly diagnosed cancer in the world with its incidence rising constantly (1). As a mainstay treatment, radiation therapy benefits approximately 50% of cancer patients and contributes to around 40% of curative cases (2). Recently, volumetric modulated arc therapy (VMAT) has been widely applied to clinical radiotherapy for its significant advantages in dose modulation.

In a typical VMAT planning, the treatment planner is required to deliver lethal and homogeneous doses to the planning target volume (PTV), while minimizing the therapeutic toxicity to the organs-at-risk (OARs) (3, 4). To satisfy this complex rule, the planner is required to perform multiple rounds of parameter adjustment and optimization in a trial-and-error manner. Ultimately, the integral dose distribution map, which visually represents the dose prescribed to each organ as well as the beam angles and numbers, is obtained. However, even with the availability of the treatment planning system (TPS), this process still costs considerable manpower and an average of up to 11 hours (5). If the planner is able to obtain the high-quality dose map prior to the planning process, and takes it as an initial point for the treatment planning, the repetitions of the trial-and-error process as well as the total planning time can be significantly reduced. Therefore, researches on obtaining high-quality dose distribution maps rapidly are of great clinical significance in providing accurate and accelerated radiotherapy.

Before our work, a range of deep-learning (6–10) has been developed to predict the dose. For example, given the limited available data in clinic, Zeng et al. (10) proposed a two-phase deep transfer learning framework to predict the dose distribution for cervical cancer patients. Nguyen et al. (6) designed a hierarchically densely connected U-net to estimate the dose distribution from the CT images, the anatomic delineations, and the prescription dose for patients with head and neck (H&N) cancer. Besides, Song et al. (7) reported DeeplabV3+ which utilized CT images and anatomic contours to predict the dose for rectal cancer patients. To take full advantage of the historical patient data, Mardani et al. (8) proposed a learning empowered approach which employed a multi-task linear regression model to predict 3D dose volume for a new patient by extracting the shared features of historical patients and their tumor shapes. Based on the clinical post-optimization strategies, Zhong et al. (9) designed a new automatic radiotherapy planning strategy that was able to produce clinically acceptable dose distributions.

More recently, generative adversarial networks (GANs) have attracted much attention from researchers due to their impressive performance in synthesis. To reduce the radiation of positron emission tomography (PET), Wang et al. (11) developed a 3D auto-context-based locality adaptive multi-modality generative adversarial networks model (LA-GANs) to synthesize the high-quality PET image from the low-dose one. Taking the advantage of U-net, Wang et al. (12) also proposed a 3D U-net-like deep architecture, combing hierarchical features by skip connections to generate full-dose PET images. Moreover, Luo et al. (13) presented AR-GAN, utilizing an adaptive rectification based generative adversarial network with spectrum constraint for standard-dose PET estimation. GAN has also made a quantum leap in the medical segmentation task. To be specific, Shi et al. (14) innovatively proposed an adaptive-similarity-based multi-modality feature selection method for Alzheimer's disease classification. Perceiving the difficulty in medical data acquisition, Wang et al. (15) proposed a triple-uncertainty guided semi-supervised model for medical image segmentation. Inspired by them, several GAN-based methods (16–18) were also proposed for dose prediction. Based on the generative adversarial network, Zhan et al. (19) developed a multi-constraint dose prediction model, capturing both global and local contextual information to predict the dose distribution for cervical and rectal cancer patients. To further reduce the time for contour delineation in radiotherapy planning, Li et al. (20) presented a multi-task attention adversarial network, including a main dose prediction task to generate the dose maps and an auxiliary segmentation task to automatically provide additional tumor delineation. Never, the developed deep-learning-based radiotherapy dose prediction models generally demand extra inputs (e.g., delineations of PTV and OARs) to supplement essential anatomical information for satisfactory predictions. Perceiving the time-consuming fact of manual contour delineation, we suggest accelerating the planning by taking the original CT images as unique input to produce high-quality dose predictions.

In order to investigate the feasibility of this CT-only dose prediction idea, we present a GAN-based framework for automatic dose prediction, which is free of manual delineation. To compensate for the missing anatomic information, we incorporate multi-task learning (MTL) into the framework by employing an auxiliary tumor segmentation task to provide essential guidance (i.e., anatomic structure of the tumor) to the primary dose prediction task. This model was trained, validated and evaluated on a cohort of 130 rectal patients. To the best of our knowledge, this is the first work that explores to predict the dose distribution maps *via* only the original CT images while mining the anatomic information automatically and concurrently.

## Materials and Methods

### Patient Cohort and CT Images

A total number of 130 postoperative patients with rectal cancer were included in this study. Ethical approval was granted by the local ethics committee. The collection of CT images followed the standard medical procedures. To be specific, each patient was immobilized with an individualized thermoplastic mask in the supine position with arms raised above the head. The intravenous contrast-enhanced CT covering the total pelvis volume for each patient was obtained. All patients were asked to drink 0.5 liter of water 1.5 h prior to scanning and refraining from voiding (21). In addition, each patient was accompanied by a PTV and four OARs including bladder, left femoral head (FHL), right femoral head (FHR), and small intestine (SI). The PTV and OARs on CT slices were delineated by the physicians based on the guideline (22) and reviewed by the senior radiation oncologists. The prescription dose of the PTV was 50.40Gy/28 fractions. The intensity-modulated radiation therapy (IMRT) treatment plans were required to cover ≥98% of the PTV with ≥93% of the prescribed dose, ≤10% of the PTV with ≥105% of the prescribed dose, and ≤5% of the PTV with ≥115% of the prescribed dose. Small intestine dose was limited to V35 <180 cc, V40 <100 cc, and V45 <65 cc. Femoral head dose was limited to V40 <40%, V45 <25%, and a maximum dose of 50 Gy. Bladder dose was limited to V40 <40%, V45 <15%, and a maximum dose 50 Gy (23). All the OARs and other normal tissues should be given as low a dose as possible. All the manual VMAT plans were conducted on the Raystation v4.7 TPS with the model for the Elekta Versa HD linear accelerators. Two 360 coplanar arc beams consisting of 91 control points respectively sharing the same isocenter were employed with 6-MV photon energy. The collimator angle was fixed at 0 and the maximum field size was 40×40 cm^2^ with a dynamic multi-leaf collimator and automatically tracking collimator jaws for each control point. Dose engine algorithm was collapsed cone convolution with the grid resolution 0.3×0.3×0.3 cm^3^. The optimization engine was the direct machine parameter optimizer with a maximum of 80 iterations and 20 iterations before conversion. The VMAT plans were completed by the senior radiation dosimetrists. All the plans were tweaked repeatedly in a trial-and-error manner until no significant improvement was found in the subsequent adjustments and optimization.

Herein, we randomly selected 98 patients for training, 10 for validation, and the remaining 22 patients for testing. Each 3D CT image was sliced into multiple 2D images with a resolution of 512×512 and a thickness of 3mm beforehand. In this manner, the training, validation, and testing samples were increased from 98, 10, and 22 to 14817, 1529, and 3491, respectively. The characteristics of these patients regarding sex and age are summarized in **Table 1**.

**Table 1:** Patient characteristics.

Characteristic		Entire Cohort (n = 130)
Sex	Male	87
	Female	43
Age	Median (IQR)	57
	Range	29-79
	≤40y	6
	40-60y	64
	≥60y	60

### Network Architecture

Generative adversarial networks (GANs) (24) have been extensively studied in computer vision over the past few years (25). As the name implies, GAN consists of a generator network and a discriminator network which are typically implemented by two independent neural networks (26). The success of GAN lies in the use of adversarial training. Specifically, in our task, the generator takes CT images as the unique input and outputs the dose distribution maps as well as anatomical structures of PTV, while the discriminator inputs the predicted dose maps and the ideal dose maps, i.e., the dose maps that were manually produced by the senior radiation dosimetrists. The goal of the generator is to produce dose maps that are too realistic to be differentiated by the discriminator, while the discriminator is trained to distinguish the generated dose maps from the real ones (also regarded as ground truth (GT)). After repeated iterations, both networks will reach Nash equilibrium, and the generator will eventually be capable of generating realistic dose maps.

### Generator Network

**Figure 1:**
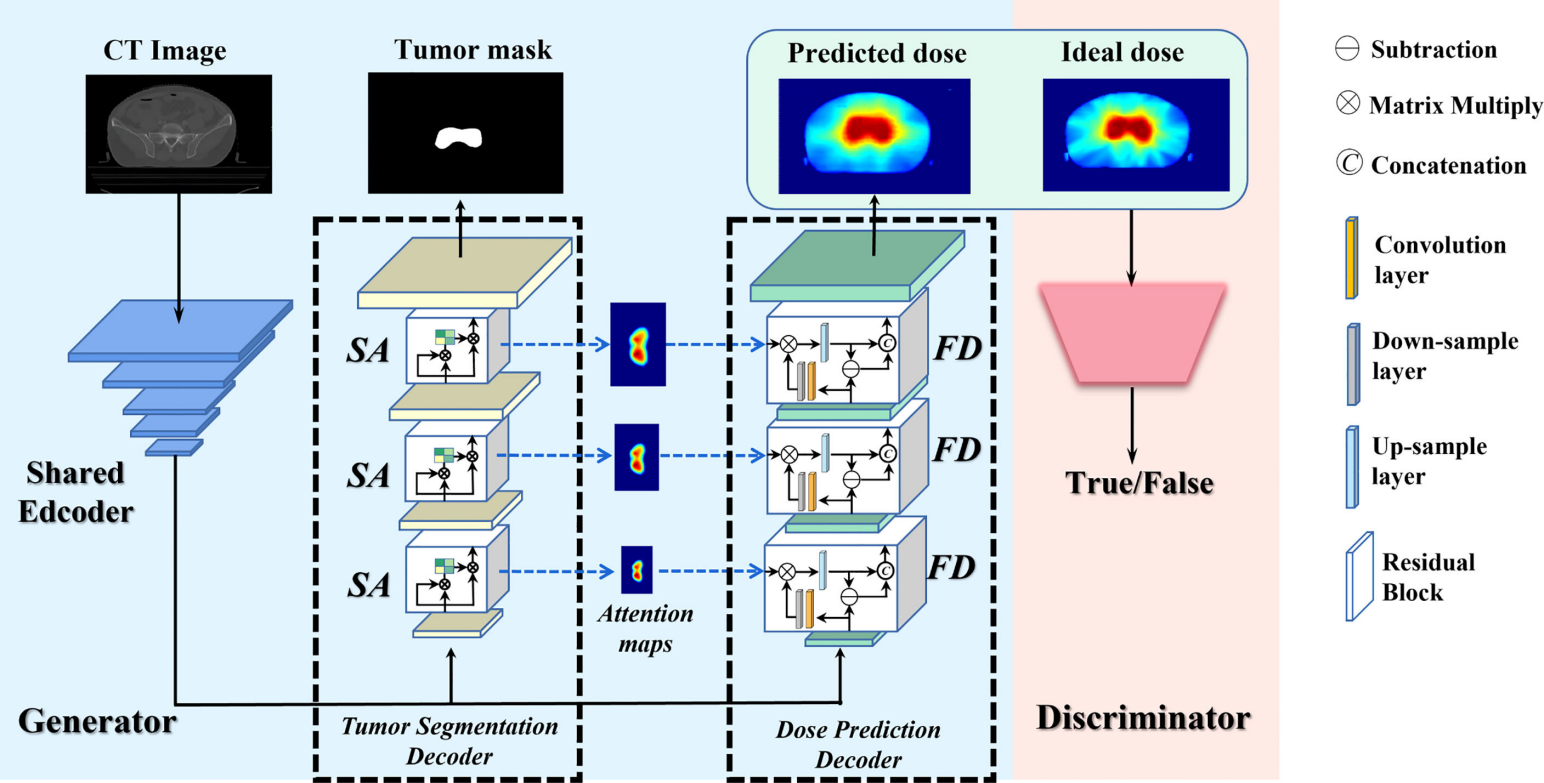
Architecture of the proposed CT-only automatic dose prediction model.

The generator is based on a U-net-like encoder-decoder architecture to integrate the shallow and deep features (27). As shown in **Figure 1**, it comprises of two tasks, i.e., the primary dose prediction task and the auxiliary tumor segmentation task, which forms the multi-task learning (MTL) architecture. To be specific, the two tasks share the same encoder and have independent decoders. The shared encoder is harnessed for semantic feature extraction from the input CT images. The dose prediction decoder aims at generating high-quality dose maps. The tumor segmentation decoder is to provide the essential anatomic structure for the dose prediction task. It is linked to the encoder and is trained synchronously with the dose prediction decoder. Particularly, we employ the self-attention (SA) module (see **Supplementary Figure A1**) in the tumor segmentation decoder, which is conducive to obtaining more precise tumor segmentation results. Each SA module outputs an attention map indicating the anatomic information (i.e., the location and shape structure) of the tumor. We transfer these attention maps to the dose prediction decoder to provide vital anatomic information of the target tumor for the primary dose prediction task. Moreover, given that an ideal dose distribution map ought to prescribe a high dose coverage in the tumor area while minimizing the dose prescription to other healthy tissues, we further design a feature decoupling (FD) module (see **Supplementary Figure A2**) in the dose prediction decoder to decouple the high- and low-dose features for the target tumor and OARs, respectively, making the model focus on the tumor and OARs separately, so that more semantically explicit dose predictions can be generated.

### Discriminator Network

The discriminator takes the predicted and the real dose distribution maps as input and tries to discriminate them correctly, which further promotes the authenticity of the predicted dose distribution map. Herein, the discriminator is implemented with a typical ResNet18 (28) architecture, which consists of seventeen convolutional layers and a fully connected layer. Each convolutional layer is followed by the BatchNormalization and ReLU activation.

### Loss Function and Network Training

The whole network is trained and optimized by a comprehensive loss function which mainly comprises two parts: a generator loss *L_G_* and a standard discriminator loss *L_D_*. The generator loss is further decomposed into two task-specific losses, i.e., a binary cross entropy loss for the tumor segmentation task, and a mean square error loss for the primary prediction task. More detailed description of the loss function is given in the **Supplementary Material**.

The proposed network was implemented using Pytorch and trained on a NVIDIA GeForce RTX 3090 GPU with 24GB memory. The batch size was set to 20. The learning rate was initialized to 5E-4 and decayed to 5E-5 linearly. The generator and the discriminator were trained alternatively using SGD optimizer for 150 epochs. To provide rich anatomic information for dose prediction task, we optimized the segmentation performance at the initial training stage by adjusting the hyper-parameters.

### Evaluation

The model with the best performance in the validation was selected for the final test. According to clinical requirements and suggestions of oncologists and dosimetrists, we adopted metrics for both PTV and OARs dose coverage, including D95 (8), average dose (Dmean), maximum dose (Dmax), conformity index (CI) (29), and homogeneity index (HI) (3). The dose-volume-histogram (DVH) curves were plotted to display the disparity between the real dose maps and the predicted dose maps intuitively. To prove the necessity and effectiveness of the key components of our method (i.e., SA module and FD module), we conducted experiments to visualize attention features of SA and FD modules. In addition, we calculated the average prediction error Δ ( $\Delta =\frac{1}{n}\sum_{i=1}^{n}\frac{\left|Prediction_{i}-GT_{i}\right|}{D_{pi}}$ , where D*_pi_*, *Prediction_i_* and *GT_i_* denote the prescribed dose, the predicted dose, and the clinically approved dose of *i*-th patient, respectively) of D95 and Dmean, denoted as ΔD95 and ΔDmean, to quantify the disparities between the predicted dose maps and the ground truths. Moreover, to verify the superiority of our prediction, we compared our proposed method with four mainstream methods, including 3D DoseNet (3), DeepLabV3+ (7), U-net (29), and GAN (16). For fair comparison, the inputs of all methods were set to be consistent, i.e., only the CT images. To further verify the clinical potential of our proposed method, we compared our predicted dose maps to DoseUnet (30), the inputs of which include both the CT images and contours. Finally, we evaluated the performance on the entire testing cohort and gave a visual summary with respect to Dmax and Dmin of ROIs in two box plots.

## Results

### Comparison With the State-of-the-Art Methods

**Figure 2:**
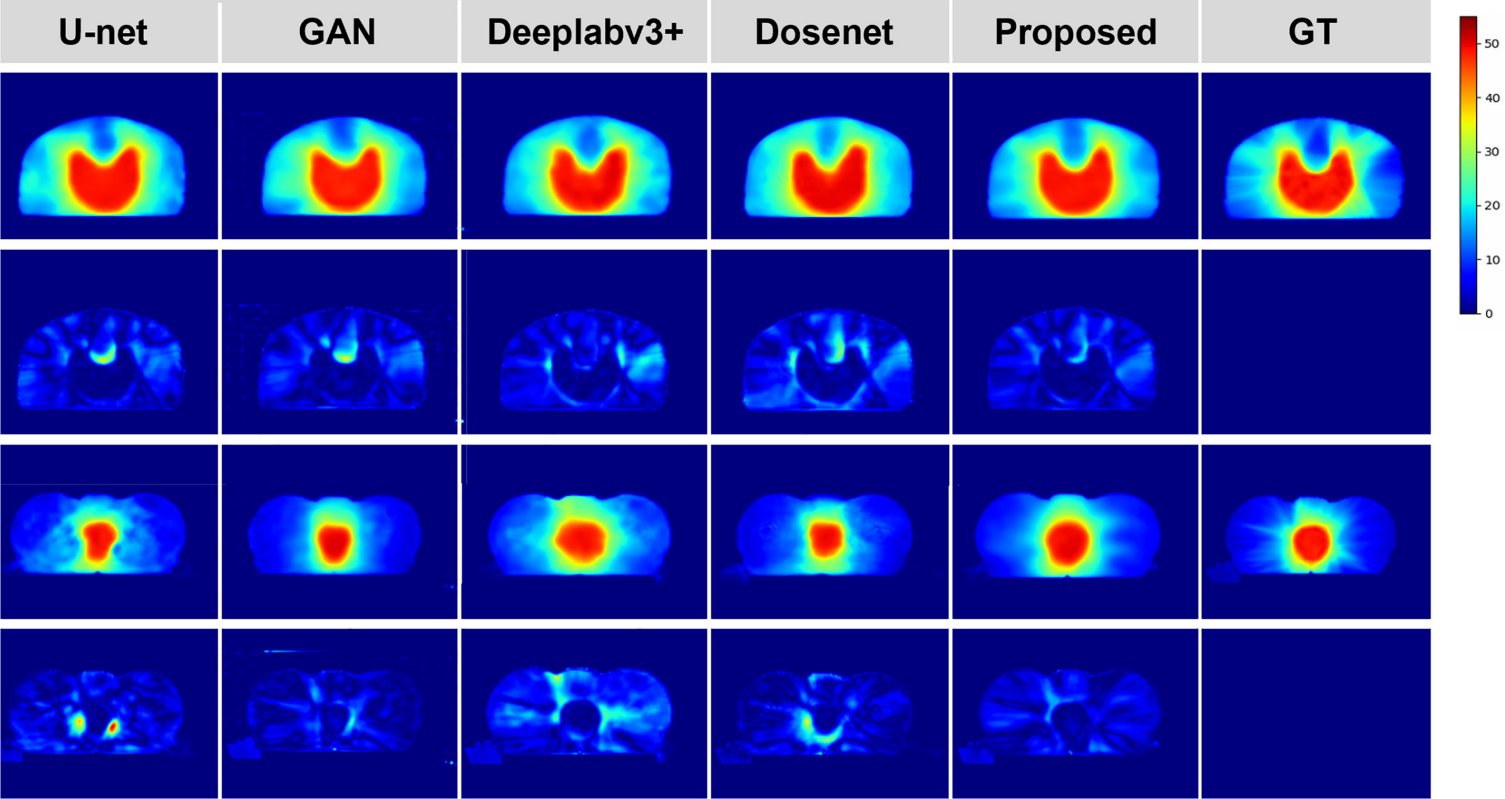
Qualitative comparison between the predicted dose distributions for the proposed and four mainstream methods. The left four columns are the dose prediction results of the comparison methods, and the right two columns are the predicted dose distributions by our method and the ground truth, respectively. The second and fourth rows of the left five columns are the difference maps calculated by subtracting the ground truth distribution map from the predicted one.

To justify the feasibility and superiority of our method, two qualitative examples predicted by our proposed method are presented and compared with the four state-of-the-art dose prediction methods in **Figure 2**. Although all of these models produce visually sound predictions, our method yields the best effect with an obvious scattering shape and a minimum error near the PTV. To provide more intuitive illustrations, we also calculate and display the difference maps. As shown in the second and fourth rows in **Figure 2**, the difference between the predicted dose of our method and the ground truth one in the clinic is the smallest with the lightest color for both PTV and OARs, indicating that our model could deliver the appropriate dose not only to the target tumor but also to the OARs.

**Figure 3:**
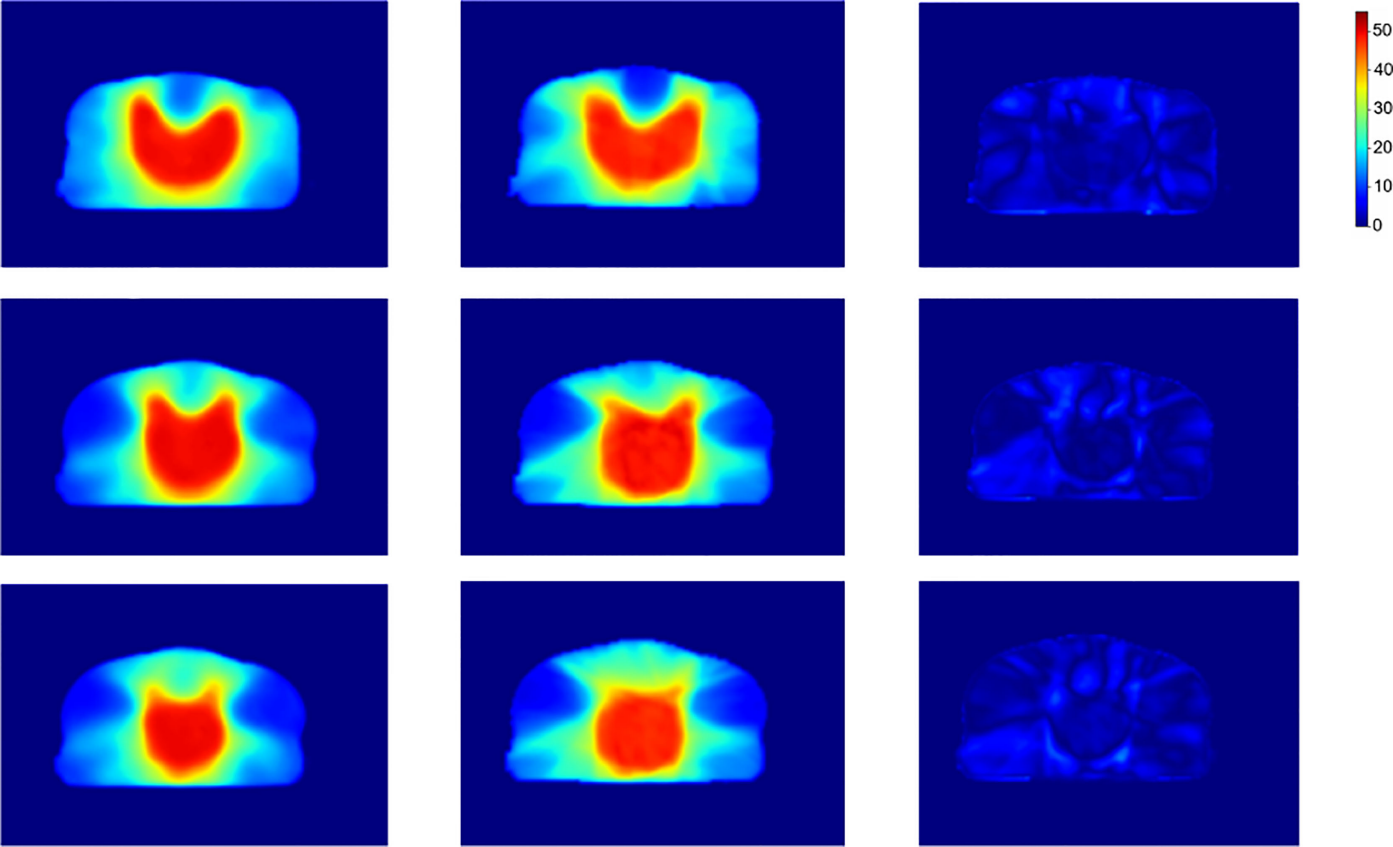
Dose distribution of our proposed method, the ground truth and the corresponding difference. From left to right shows the slices predicted by our method, the corresponding slices of the ground truth, and the dose difference, respectively.

**Figure 4:**
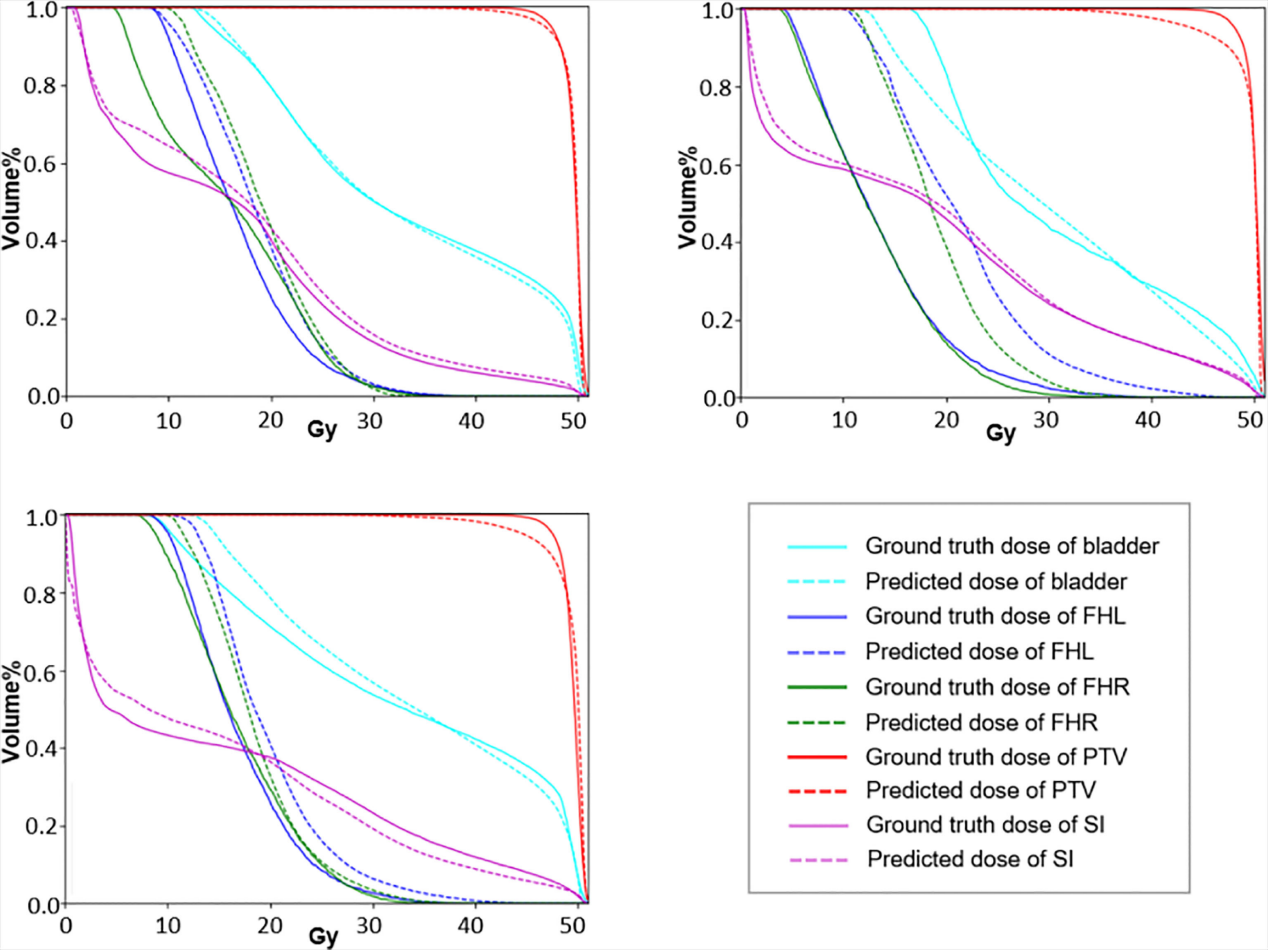
The DVH curves of three typical predictions. The dotted lines represent the prediction results and the solid lines represent the approved values.

To further demonstrate the effectiveness of our method, we present a set of qualitative examples produced by our model in **Figure 3** and compare them with their corresponding ground truth. According to the dose difference map displayed in the third column, it is evident that our CT-only method could produce dose distribution close to the ground truth in all cases, especially in the target tumor area. Besides, three typical DVH examples are displayed in **Figure 4**. The differences between the predicted dose curves and the ground truth ones are subtle, especially for the PTV whose disparities are minimal.

**Figure 5:**
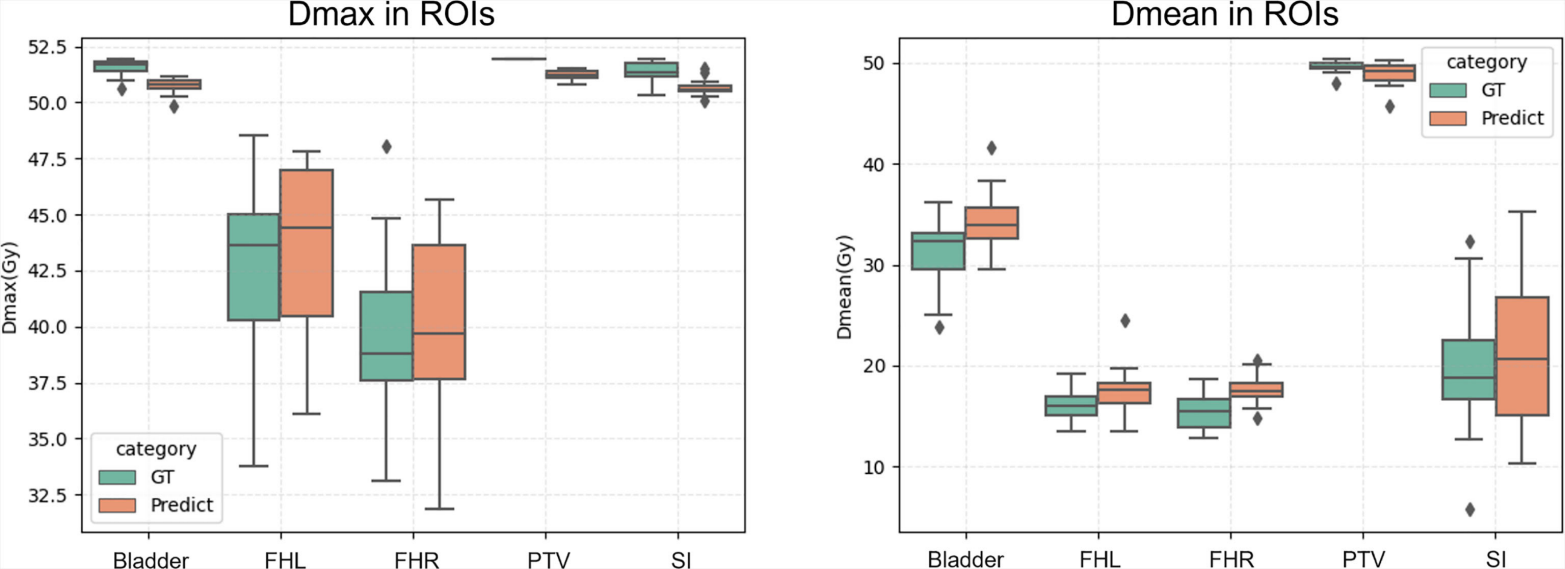
Dmax (left) and Dmean (right) of the proposed prediction and the ground truth with respect to ROIs. Horizontal lines in boxes are medians and rhombuses are outliers.

In Addition, a visual summary of our predicted dose and the clinically acceptable dose with respect to Dmax and Dmean of ROIs are illustrated in **Figure 5**. By comparing the respective median and data dispersion of each box plot, one can see that our predicted dose maps share the same dose distribution with the clinically approved ones in terms of Dmax and Dmean. Specifically, our predicted results had the same skewness with the approved dose in Dmax with respect to FHL and FHR.

The results of quantitative comparison are given in **Table 2**. Our method outperforms other mainstream dose prediction methods by achieving the best results in three out of four metrics with the highest HI of 1.023 (3.27E-5) and the lowest prediction error, i.e., ΔD95 of 0.125 (0.035) and ΔDmean of 0.023 (4.19E-4), respectively. Particularly, with the best HI index, our method is capable of producing homogeneous dose distribution in PTV, and the lower errors in D95 and Dmean also meet the clinical expectation. In addition, the paired t-test was conducted, and the p-values are less than 0.05 in most of the evaluation metrics, demonstrating the significant improvements of our method. Moreover, we also compared our proposed method with DoseUnet which is aided by the additional tumor and OARs contours. The results are shown in the fifth row of **Table 2**. In terms of HI and ΔDmean, our method achieves comparable performance with DoseUnet.

**Table 2:** Quantitative comparisons with four mainstream dose prediction methods in terms of HI, CI, D95, and Dmean.

Method	HI	CI	Average prediction error ↓	
			ΔD_95_	ΔD_mean_
U-net (26)	1.013 (4.41E-6)*	0.598 (0.006)*	0.301 (0.074)*	0.044 (1.12E-3)*	
DeepLabV3+ (7)	1.022 (7.53E-6)	0.593 (0.005)*	0.269 (0.048)*	0.038 (1.16E-3)*	
DoseNet (3D) (3)	1.019 (9.68E-6)*	0.592 (0.009)*	0.211 (0.055)*	0.035 (1.11E-3)	
GAN (16)	1.016 (2.86E-5)*	0.626 (0.007)	0.204 (0.061)*	0.038 (8.35E-4)*	
DoseUnet (30)	1.013 (3.82E-5)	0.736 (0.006) ^¶^	0.071 (0.047) ^¶^	0.027 (6.00E-3)	
Proposed	1.023 (3.27E-5)^¶^	0.624 (0.009)	0.125 (0.035)	0.023 (4.19E-4)^¶^	

The HI, CI, ΔD_95_, and ΔD_mean_ are displayed in the form of mean (variance). The ground truth of HI and CI are 1.030 and 0.773, respectively. Please refer to Evaluation section for more details of the definition.

*Our method is significantly better than the compared ones, i.e., p < 0.05 via paired t-test.

^¶^The best results of each index.

↓The lower the average prediction error is, the better the dose prediction result is.

### Ablation Study

The key components of our method include 1) the auxiliary tumor segmentation task (Aux), 2) SA module, 3) discriminator (Disc), and 4) FD module. To investigate the contributions of these components, we further conducted a series of ablation experiments with following variants: (1) U-net with residual blocks embedded alone (i.e., Baseline), (2) Residual blocks embedded U-net with the auxiliary segmentation module (i.e., Baseline+Aux), (3) The network in (2) with SA module embedded (i.e., Baseline+Aux+SA), (4) The network in (3) with the discriminator (i.e., Baseline+Aux+SA+Disc), (5) The network in (4) with FD module injected (Baseline+Aux+SA+Disc+FD, proposed). The quantitative results are given in **Table 3**. We can clearly see that the performance of the network improves progressively as each component is added to the Baseline framework.

**Table 3:** Ablation studies of our propose method with its variants.

Method	DSC↑	HI	CI	Average prediction error ↓	
				ΔD_95_	ΔD_mean_
(1) Baseline	–	1.013 (1.60E-5)*	0.615 (0.008)	0.238 (0.052)*	0.040 (1.12E-3)*
(2) Baseline+Aux	0.802 (0.002)*	1.019 (1.18E-5)*	0.584 (0.008)*	0.208 (0.051)*	0.038 (8.35E-4)*
(3) Baseline+Aux+SA	0.809 (0.003)*	1.017 (2.75E-5)*	0.625 (0.010)	0.161 (0.032)	0.033 (5.74E-4)*
(4) Baseline+Aux+SA+Disc	0.815 (0.003)	1.020 (9.65E-6)*	0.629 (0.008)^¶^	0.162 (0.038)	0.031 (5.36E-4)*
(5) Baseline+Aux+SA+Disc+FD (Proposed)	0.816 (0.003)^¶^	1.023 (3.27E-5)^¶^	0.624 (0.009)	0.125 (0.035) ^¶^	0.023 (4.19E-4)^¶^

↑The higher the DSC is, the better the dose prediction result is.

**Figure 6:**
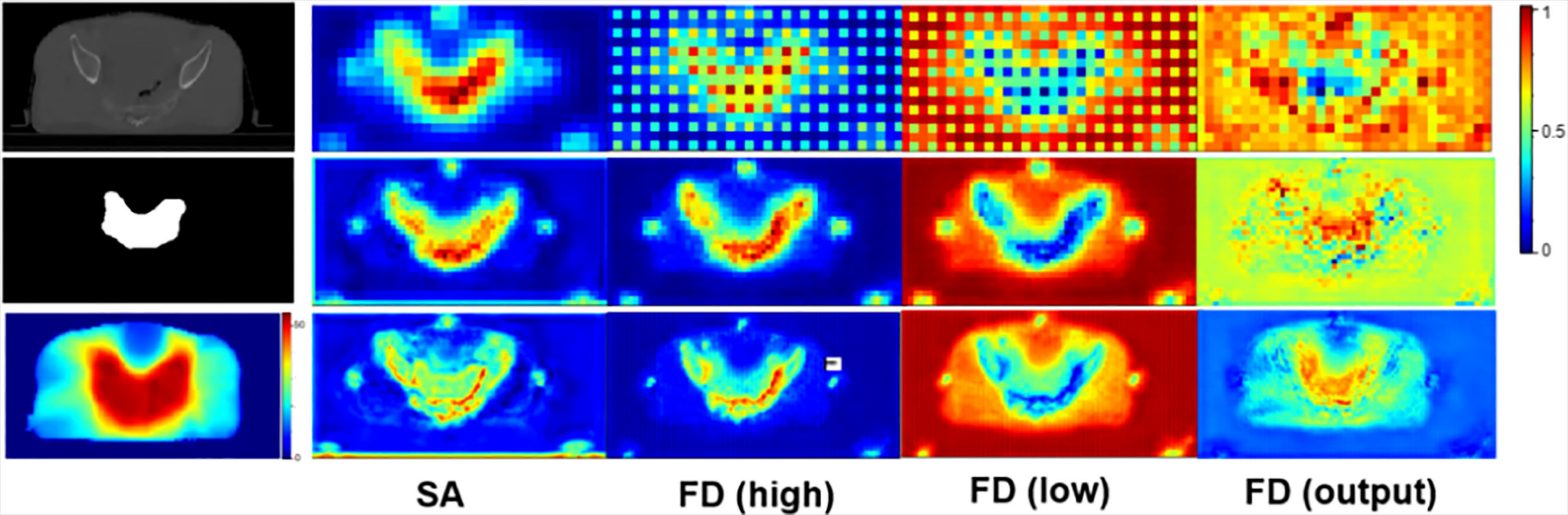
The attention-weighted feature maps for SA and FD. The first column from top to bottom shows the input CT image, tumor segmentation and dose distribution map separately. The second column visualizes the attention of SA module in tumor segmentation task. The following three columns illustrate the attention of FD module in dose prediction task. Specifically, FD (high) and FD (low) denote high- and low-dose features, respectively, and the refined output of FD is marked as FD (output). The redder the area is, the more attention the network pays. The top row refers to the results of the shallowest layer while the bottom row stands for the results of the deepest layer.

To further investigate the effectiveness of our SA and FD modules, we visualize the attention-weighted feature maps for these two modules in both the segmentation decoder and the dose prediction decoder. The results are shown in **Figure 6**. After the SA module, most attention is delivered to the tumor region, thus demonstrating the positive effect of this module on tumor segmentation. In addition, the third and the fourth columns, i.e., FD (high) and FD (low), focus on the tumor area and the opposite healthy tissues, respectively, proving the capability of our network in decoupling high- and low-dose features.

## Discussion

In this paper, we undertook an exploratory study to investigate the feasibility of producing high-quality radiotherapy dose predictions *via* only the original CT images, i.e., being free of manual delineation of PTV and OARs.

Two main steps in radiotherapy planning that require considerable manpower and resources are manual contour delineation and repeated parameter tweaking (7). A large number of deep-learning-based dose prediction models have achieved promising results with the assistance of manual delineations of PTV and OARs. The predicted high-quality dose distribution maps can be introduced as a guidance tool and an initial point to improve the efficiency of the inverse parameter adjustment. However, researches indicated that the average time for manual contouring can be up to 3 hours, which may lead to a delay in the treatment and induce errors in tumor localization (16). Current deep-learning-based methods pay little attention on reducing the time spent for manual delineation. In view of this, we boldly suggested predicting the dose maps based solely on the original CT images to further reduce the total planning time by incorporating automate delineation. Concretely, we proposed a GAN-based model and adopted the MTL strategy to learn the missing anatomic contours of the tumor, thereby guiding the primary dose map prediction task.

Our CT-only method was trained on a cohort of 130 rectal cancer patients and evaluated on metrics of D95, Dmean, Dmax, CI, and HI to study the dosimetric congruence between the predicted dose distributions and the approved ones. DVH curves were also plotted for comparison between the approved dose maps and our predicted dose maps. We have compared our method with four mainstream dose prediction methods with contours input removed in **Table 2**. As observed, compared to the widely used U-net model, our method considerably improves the prediction with HI and CI rising from 1.013 (4.41E-6) to 1.023 (3.27E-5), and from 0.598 (0.006) to 0.624 (0.009) respectively. Meanwhile, one can see that the prediction errors ΔD95 and ΔDmean drop from 0.301 (0.074) to 0.125 (0.035), and from 0.044 (1.12E-3) to 0.023 (4.19E-4), respectively. Additionally, compared to DeepLabV3+, our method also improves CI, ΔD95, and ΔDmean by 0.031, 0.144, and 0.015 respectively. Furthermore, the proposed method still surpasses GAN by 0.007 HI, 0.079 ΔD95, and 0.015 ΔDmean, respectively. Although not achieving the best value, the CI acquired by our method (i.e., 0.624) approximates the optimal one (i.e., 0.626) with a minor and tolerable difference of 0.002. Besides, our proposed model could achieve a general minimal difference between the whole testing cohort and the ground truth with respect to Dmax and Dmean of ROIs, as shown in **Figure 5**, manifesting its potential clinical application. To further validate the practicability of our proposed method, we compared our predicted dose maps with those of a contour-aided method, i.e., DoseUnet. As illustrated in **Table 2**, we noticed that the proposed CT-only dose prediction method could achieve comparable results to DoseUnet on both HI and ΔDmean metrics. As for the other metrics, for example, ΔD95 and CI, we must admit that blocking the prior accurate anatomical information in the input will inevitably bring performance degradation. However, this does not mean that our method loses its clinical significances and values. Actually, studies show that the time of contouring is usually longer than that of dose calculation in clinical practice (5), so the saving time in manual delineation brought by our work would be longer than the added tuning time in case of little performance decrease. We will make quantitative comparison when conditions permit.

To investigate the contribution of each module in the proposed network, we conducted a series of ablation study in **Table 3**. Firstly, by comparing (1) and (2), we demonstrated the ability of the tumor segmentation task in providing essential anatomical information for the dose prediction task. As can be seen, with the assistance of the auxiliary task, the HI, ΔD95, and ΔDmean are improved by 0.006, 0.03, and 0.002, respectively. Notably, given the similarities between the SA and FD (high), we can see that the anatomic information obtained in the tumor segmentation task has been successfully transferred to the dose prediction task as guidance. Secondly, we validated the potency of the embedded SA module by comparing (2) and (3). It’s clear that the proposed SA module improves the dose prediction performance by 0.041 CI, 0.047 ΔD95 and 0.005 ΔDmean, respectively. Thirdly, in order to verify the contribution of the discriminator in the dose prediction task, we compared (3) and (4). After adding the discriminator, there are also more or less improvements in DSC, HI, CI and ΔDmean. Finally, we compared (4) and the complete model (5) to verify the usefulness of the proposed FD module.

Furthermore, we visualized the attention maps of FD (high) and FD (low) in **Figure 6**. Accordingly, the opposite attention of FD (high) and FD (low) demonstrated the success of our method in decoupling high- and low-dose features. Meanwhile, the increasing tendency of attention in both high- and low-dose areas, as shown in the output of FD, i.e., FD (output), further manifested the capability of our method in paying attention to the high dose distribution in the tumor region while not neglecting the low dose distribution in the surrounding healthy tissues. This simultaneous attention on both the tumor and the surrounding tissues contributed to a more accurate radiotherapy planning.

Despite the superior performance of our method, there are still some limitations. Firstly, our current method employs 2D images due to limited computational resource, which may ignore the geometry information of the organs. In the future, we will extend our dose prediction method to a 3D model to improve the exactness of the anatomic information. Secondly, the auxiliary segmentation task only considers tumor segmentation. However, the anatomic information of the surrounding organs, i.e., OARs, is also of great clinical significance and is critical for the dose prediction. On this basis, we will extend our single-target segmentation model to a multi-target one (i.e., implementing the segmentation on every organ in the CT image), thus providing organ-specific radiation dose prediction. Thirdly, the sub-optimal quality of the generated contours may impede the generation of realistic dose distributions. Given the possible inaccuracy that lies in the essential guiding contours, we will search for a more accurate contour segmentation method in the future.

It is worth mentioning that the generated dose map is regarded as a guidance tool for dosimetrists to reduce the manual intervention and lead a more accurate and speedy treatment planning rather than to provide the ultimate solutions, i.e., the final treatment plan to be performed in the clinic. In other words, the treatment planners still need to adjust the radiotherapy parameters manually but with much reduced time and effort. The goal of our method is to quicken the radiotherapy planning by providing high-quality dose distribution map, which not only brings an initial point close to the optimal plan (instead of presetting the parameters empirically) but also presents a possible optimal target for parameter adjustment.

## Conclusion

This exploratory study proves the feasibility of predicting high-quality dose distribution with only CT images. Albeit omitting the manual delineations of critical organs, this model introduces a well-designed MTL strategy to make up for the missing anatomic information. Experimental results manifest the capability of the proposed CT-only dose prediction model in producing more realistic dose predictions for rectal cancer radiation therapy compared to the mainstream contour-aided ones.

## Data Availability Statement

The datasets generated and/or analyzed during the current study are not publicly available due to data security but are available from the corresponding author on reasonable request.

## Ethics Statement

The studies involving human participants were reviewed and approved by West China Hospital, Sichuan University. The patients/participants provided their written informed consent to participate in this study.

## Author Contributions

ZJ and YW contributed to the conceptualization of this project. ZJ contributed to the investigation, methodology, and software. JX and XP provided the resources and data. JC drafted and ZJ, YW, and JX reviewed and edited the manuscript. JX, ZW, XH, and XP provided expert clinical knowledge. YW supervised and administrated the project. All authors contributed to the article and approved the submitted version.

## Funding

This work is supported by National Natural Science Foundation of China (NSFC 62071314) and Sichuan Science and Technology Program (2021YFG0326, 2020YFG0079).

## Conflict of Interest

The authors declare that the research was conducted in the absence of any commercial or financial relationships that could be construed as a potential conflict of interest.

## Publisher’s Note

All claims expressed in this article are solely those of the authors and do not necessarily represent those of their affiliated organizations, or those of the publisher, the editors and the reviewers. Any product that may be evaluated in this article, or claim that may be made by its manufacturer, is not guaranteed or endorsed by the publisher.
