# Supplementary material for: CT-Only Radiotherapy: An Exploratory Study for Automatic Dose Prediction on Rectal Cancer Patients Via Deep Adversarial Network
Source: Front Oncol. 2022 Jul 18;12:875661. doi: 10.3389/fonc.2022.875661 (PMC9341484; doi:10.3389/fonc.2022.875661)
Supplement: Supplementary file 1 [file DataSheet_1.docx]

Supplementary Material

# Supplemental Network Architecture Diagrams

As shown in Fig.1 of this paper, we devised a standard self-attention module (SA) for the tumor segmentation decoder to make the auxiliary decoder pay more attention to the tumor area. The detailed architecture is shown in Fig.A.1. Specifically, we applied a 1×1 convolution to the intermediate 2D feature maps of size h×w (h and w denote the height and width of feature maps respectively) with the number of c and obtained the query(q), key(k) and value(v) of size [c, h, w]. Then, by performing matric multiplication on q and k, the attention map of size [h×w, h×w] was obtained. Finally, the multiplication of the attention map and value (v) was performed as the output of the SA module.


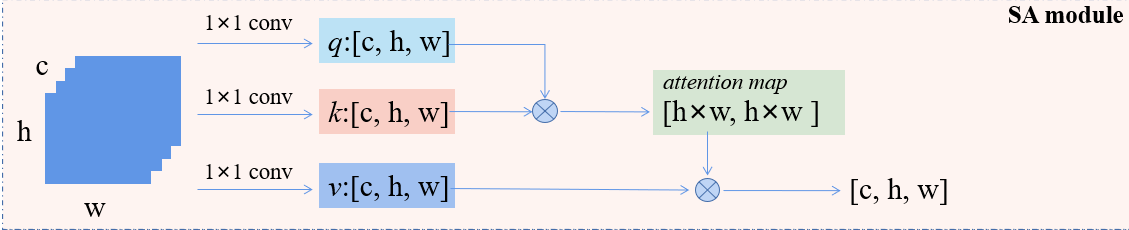


**Supplementary Figure A1.** The detailed architecture of the self-attention module.

For the primary dose prediction task, both the tumor and its surrounding tissues should be delivered an appropriate dose distribution. Therefore, the primary task is encouraged to pay attention to both the tumor and its surrounding OARs. To achieve this, we design a feature decoupling (FD) module and embed it between each two residual blocks in the primary dose prediction decoder. As displayed in Fig.A2, the FD module input two parts including: 1) the output of the *i*-th convolution block of dose prediction decoder $f_{i}$ and 2) the attention map ${atten}_{i}$ learned by the corresponding SA module of the segmentation decoder, and output the fused feature $f_{i}^{out}$. To obtain a new feature map $f_{i}^{h}$ whose attention was focused on the tumor area with high-dose distribution, we performed a 2D convolution and two-stride down-sample on $f_{i}$ following a matric multiplication with ${atten}_{i}$ which was reduced to half of its original size by the down-sample layer. Then, $f_{i}^{h}$ was obtained after a 2D up-sampling operation of the product. It needs to be stated that the up-sampling and down-sampling used in FD was only for the purpose of reducing the computational consumption related to the attention map. After that, to pay attention to the low-dose OARs-area simultaneously, we inputted the difference map $f_{i}^{d}$ which was obtained by subtracting $f_{i}^{h}$ from $f_{i}$, into a SA module, producing meaningful feature $f_{i}^{l}$ that focused on the low-dose coverage. In this manner, both high- and low-dose distribution area, represented by $f_{i}^{h}$ and $f_{i}^{l}$, were decoupled successfully. Finally, $f_{i}^{h}$ and $f_{i}^{l}$ are concatenated and passed through a 3×3 2D convolutional layers to obtain the fused feature $f_{i}^{out}$.

**Supplementary Figure A2.** The detailed architecture of the feature decoupling module.

# Supplemental Mathematical Derivation Process of the Optimization Function

For the tumor segmentation loss, we employ the commonly used binary cross entropy (BCE) loss to minimize the gap between the predicted segmentation mask $y_{s}^{'}$ and the corresponding ground truth $y_{s}$. The formula is shown as follows:

$L_{seg}\left( y_{s},y_{s}^{'} \right)=E\left[ -\left( y_{s}log(y_{s}^{'})+\left( 1-y_{s} \right)log\left( 1-y_{s}^{'} \right) \right) \right]$ Eq.(B.1)

where $y_{s}^{'}$ is the predicted segmentation mask and $y_{s}$is the corresponding ground truth.

For the dose prediction loss, the L1 loss is employed to encourage the similarity between the predicted dose distribution map $y_{d}^{'}$ and the real dose distribution map $y_{d}$. An additional L2 loss further reduces the gap between the predicted dose distribution of the tumor $y_{tum}^{'}$ and to the real $y_{tum}$. The formula is denoted as follows:

$L_{dose}=L_{L1}\left( y_{d},y_{d}^{'} \right)+L_{L2}\left( y_{tum},y_{tum}^{'} \right)$ Eq.(B.2)

where $L_{L1}\left( y_{d},y_{d}^{'} \right)=E\left[ \left\| y_{d}-y_{d}^{'} \right\|_{1} \right]$ and $L_{L2}\left( y_{tum},y_{tum}^{'} \right)=E\left[ \left\| y_{tum}-y_{tum}^{'} \right\|_{2} \right]$, $y_{tum}$ and $y_{tum}^{'}$ can be derived by multiplying $y_{s}$ with $y_{d}$ and $y_{d}^{'}$, respectively.

We also employ a novel adversarial loss to capture the high-frequency texture information and avoid blurring results. Moreover, this adversarial loss can improve the distribution authenticity of the predicted dose maps. The objective function of the generator G is defined as:

$L_{adv}^{G}=- E\left[ \log D\left( y_{d}^{'} \right) \right]$ Eq.(B.3)

where D is the discriminator.

The overall generator loss is expressed as:

$L_{G}=\lambda_{seg}L_{seg}+\lambda_{dose}L_{dose}+ {\lambda_{adv}L}_{adv}^{G}$ Eq.(B.4)

where λ_seg, λ_dose and λ_adv are hyperparameters to balance the three terms. In the experimental phase, λ_seg and λ_adv were set to 10 and 1 empirically. The value of hyper-parameter λ_dose was initially set to be small at the beginning and then increased 1 at each epoch from 1 to 150 gradually.

Similarly, the discriminator loss is denoted as:

$L_{D}=E[ logD(y_{d}^{'}) ]+E[ log(1-D(y_{d})) ]$ Eq.(B.5)

where D is the discriminator.
